# On the Theory and Practice of Midwifery

**Published:** 1851-11

**Authors:** 


ARTICLE II.

*On the Theory and Practice of Midwifery.* By FLEETWOOD CHURCHILL, M. D., M. R., I. A., Hon. Fellow of the College of Physicians in Ireland, Member of the American National Institute, etc. With notes and additions by D. FRANCIS CONDIE, M. D., Sec'y. Col. Physicians, Philad., etc., etc. With one hundred and thirty-nine illustrations. A new American, from the last improved Dublin edition; pages, 510 octavo. Blanchard & Lee, Philadelphia, 1851. (From the Publishers, through S. C. Griggs & Co., Chicago.)

This is a new edition of our best systematic work on Obsteterics. It is so full of accurate information, and so clear of speculation and controversy, that it has deservedly been regarded as worthy of a place at the head of the long catalogue of works upon the subject that have been issued from the American press. Our readers, however, are so familiar with it that it is not worth our while to speak of it more at length.

It is got up in the good style in which the publishers are generally careful to clothe their publications.

